# Retraction: Ajibade, P.A., et al. Synthesis, Optical and Structural Properties of Copper Sulfide Nanocrystals from Single Molecule Precursors. *Nanomaterials* 2017, *7*, 32

**DOI:** 10.3390/nano8121047

**Published:** 2018-12-14

**Authors:** Peter A. Ajibade, Nandipha L. Botha, Nanomaterials Editorial Office

**Affiliations:** 1 Department of Chemistry, University of Fort Hare, Private Bag X1314, Alice 5700, South Africa; 200904696@ufh.ac.za; 2 St. Alban-Anlage 66, 4052 Basel, Switzerland

The *Nanomaterials* Editorial Office has been made aware that the published paper [1] was previously published in *Results in Physics* [2] by the same authors. In order to preserve academic integrity, the title paper [1] will be marked as retracted. 

We apologize to the readers of *Nanomaterials* for any inconvenience caused, and regret that this issue was not detected earlier. The decision to retract has been taken in agreement with the authors. The manuscript [1] was submitted shortly after the paper had been accepted [2] in *Results in Physics*, and prior to its publication. The issue resulted from an incorrect version submitted by the authors during the editorial process. 

MDPI is a member of the Committee on Publication Ethics, and takes its responsibility to enforce strict ethical policies and standards very seriously. To ensure the addition of only high-quality scientific works to the field of scholarly publications, reference [1] is retracted and shall be marked accordingly.
